# Supplementation of Persimmon Leaf Ameliorates Hyperglycemia, Dyslipidemia and Hepatic Fat Accumulation in Type 2 Diabetic Mice

**DOI:** 10.1371/journal.pone.0049030

**Published:** 2012-11-08

**Authors:** Un Ju Jung, Yong Bok Park, Sang Ryong Kim, Myung-Sook Choi

**Affiliations:** 1 Department of Food Science and Nutrition, Kyungpook National University, Daegu, Republic of Korea; 2 School of Life Sciences and Biotechnology, Kyungpook National University, Daegu, Republic of Korea; 3 Brain Science and Engineering Institute, Kyungpook National University, Daegu, Republic of Korea; Pennington Biomedical Research Center, United States of America

## Abstract

Persimmon Leaf (PL), commonly consumed as herbal tea and traditional medicines, contains a variety of compounds that exert antioxidant, α-amylase and α-glucosidase inhibitory activity. However, little is known about the *in vivo* effects and underlying mechanisms of PL on hyperglycemia, hyperlipidemia and hepatic steatosis in type 2 diabetes. Powered PL (5%, w/w) was supplemented with a normal diet to C57BL/KsJ-*db/db* mice for 5 weeks. PL decreased blood glucose, HOMA-IR, plasma triglyceride and total cholesterol levels, as well as liver weight, hepatic lipid droplets, triglycerides and cholesterol contents, while increasing plasma HDL-cholesterol and adiponectin levels. The anti-hyperglycemic effect was linked to decreased activity of gluconeogenic enzymes as well as increased glycogen content, glucokinase activity and its mRNA level in the liver. PL also led to a decrease in lipogenic transcriptional factor PPARγ as well as gene expression and activity of enzymes involved in lipogenesis, with a simultaneous increase in fecal lipids, which are seemingly attributable to the improved hyperlipidemia and hepatic steatosis and decreased hepatic fatty acid oxidation. Furthermore, PL ameliorated plasma and hepatic oxidative stress. Supplementation with PL may be an effective dietary strategy to improve type 2 diabetes accompanied by dyslipidemia and hepatic steatosis by partly modulating the activity or gene expression of enzymes related to antioxidant, glucose and lipid homeostasis.

## Introduction

Persimmon (*diospyros kaki)* is a widely cultivated fruit in East Asia, and the persimmon leaf (PL) is commonly used for herbal tea and traditional medicines in South Korea, Japan and China. PL contains proanthocyanidins (also called condensed tannins), flavonoids and other compounds, which have a variety of pharmacological actions [1]–[7]. Flavonoids isolated from PL have antioxidant [1], hypotensive [2] and anti-allergic effects [3] and proanthocyanidins, the major polyphenol in PL, have anti-hypertensive and vasorelaxant effects [4]. Some *in vitro* studies have suggested that PL may also have beneficial effects on diabetes. For example, components from PL inhibited α-amylase, α-glucosidase and protein tyrosine phosphatase 1B activity [5]–[7], and stimulated glucose uptake in HepG2 cells and 3T3-L1 adipocytes [6]. However, there is relatively little known regarding the *in vivo* efficacy of PL for diabetes.

Type 2 diabetes, the most common type of diabetes, is one of the fastest growing and most costly metabolic disorders in the world. It is accepted that changes in lifestyle, such as increased fat intake and/or physical inactivity and decreased vegetable, fruits and whole grain intake, are responsible for the increasing the incidence of type 2 diabetes. Insulin resistance is a major underlying factor contributing to the development of type 2 diabetes, which can result in hyperglycemia, dyslipidemia, or hepatic steatosis. The liver is a key metabolic buffering organ that controls glucose and lipid homeostasis, and, in particular, hepatic insulin resistance can cause hyperglycemia through reduced glycogen synthesis and storage and a failure to suppress glucose production and release into the blood. In addition, hepatic steatosis is linked to insulin resistance, although whether the excessive accumulation of lipids in the liver is a cause or a consequence of insulin resistance still remains unclear [8]. Since hyperglycemia- and hyperlipidemia-induced reactive oxygen species is one of the major contributors of insulin resistance [9], the identification of antioxidant foods, which can improve insulin sensitivity, is crucial for the amelioration of type 2 diabetes and its complications.

We previously reported that powdered PL improved lipid profiles and suppressed body weight gain in rats fed a high-fat diet [10]. In this study, we investigated the anti-diabetic effect of PL in type 2 diabetic *db/db* mice that have an exacerbation of insulin resistance and hepatic steatosis [11]. We also examined the potential mechanisms of action, particularly focusing on the gene expression and activity of enzymes involved in glucose and lipid metabolism and oxidative stress in the liver as well as the lipid content in the feces.

## Materials and Methods

### Preparation of Powdered Persimmon Leaf and its Composition Analysis

PL was prepared as described previously [10]. PL was harvested in Sangju (Korea) and dried in the shade for a week. The leaf was powdered and passed through 60 mesh sieves. The total fiber and phenolic contents were analyzed by AOAC method and a modified Folin-Ciocaleu colorimetric method, respectively [12], [13]. For determination of total phenolic content, a solution containing extract or standard solution of gallic acid was mixed with Folin-Ciocalten reagent. After 6 min, 7% sodium carbonate solution was added to the mixture. The resulting solution was incubated for a further 90 min before absorbance reading was spectrophotometrically read at 760 nm. The total phenolic content was expressed as mg of gallic acid equivalents/g of powdered PL. The total flavonoid content was determined according to Moreno et al. [14] and was expressed as mg of quercetin equivalents/g of powdered PL. The total fiber, phenolic and flavonoid contents in the PL were 630 mg/g, 11.49 mg/g, 1.59 mg/g respectively.

### Animals and Diets

Male C57BL/KsJ-*db*/*db* (*db/db*) mice were purchased from Jackson Laboratory (Bar Harbor, ME) at 5 weeks of age and maintained under standard light (12 h light/dark) and temperature conditions (22±2°C). The twenty *db/db* mice were fed a pelletized commercial chow diet for 2 weeks after arrival, and then the *db/db* mice were divided into two groups (n = 10). Thereafter, the control group of *db/db* mice were fed a standard semisynthetic diet (AIN-76) [15], [16], while another group of *db/db* mice were fed a standard semisynthetic diet with the powered PL (5 g/100 g diet, w/w) for 5 weeks. The mice had free access to food and water *ad libitum*. At the end of the experimental period, the mice were anesthetized with ketamine after withholding food for 12 hours, and blood samples were taken from the inferior vena cava to determine the plasma biomarkers. In addition, the liver was removed after the blood was collected, then rinsed with a physiological saline solution, and immediately stored at −70°C. All procedures were approved by the animal ethics committee of Kyungpook National University (Approval No. KNU-2011-28).

### Fasting Blood Glucose Level and Homeostatic Index of Insulin Resistance (HOMA-IR)

Every week after 12 hours of fasting, the blood glucose concentration was monitored in the venous blood from the tail vein using a glucometer (Arkary, Japan). HOMA-IR was calculated according to the homeostasis of the assessment as follows (Eq. 1) [17]:



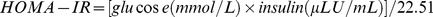
.

### Plasma Analyses

The blood was collected in a heparin-coated tube and centrifuged at 1,000×g for 15 min at 4°C. The plasma insulin and adiponectin levels were determined using an insulin RIA kit (Diagnostic Systems Laboratories, USA) and a sandwich ELISA kit (R&D system, USA), respectively. The plasma free fatty acid concentration was measured using an enzymatic non-esterified fatty acid kit (Wako, Osaka, Japan). Meanwhile, the plasma triglyceride, total cholesterol, and high-density lipoprotein (HDL)-cholesterol concentrations were measured spectrophotometrically using a commercial kit (Sigma Chemical Co.). The paraoxonase (PON) activity was assayed spectrophotometrically using the method described by Mackness et al. [18], which measured the increase in absorbance for 90 s at 405 nm and 25°C.

### Hepatic and Fecal Lipids and Hepatic Glycogen, Hydrogen Peroxide and Lipid Peroxidation

The feces from each group were collected daily for one week, and the hepatic and fecal lipids were extracted using the procedure developed by Folch et al. [19]. The levels of cholesterol and triglyceride in the liver and feces were analyzed with the same commercial kit as used in the plasma analysis.

The hepatic glycogen content was determined as previously described by Seifter et al. [20] with modification. Briefly, the liver tissue was homogenized in 5 volumes of an 30% (w/v) KOH solution and dissolved at 100°C for 30 min. The glycogen was determined by treatment with an anthrone reagent (2 g anthrone/1 L of 95% (v/v) H_2_SO_4_) and measuring the absorbance at 620 nm.

The hydrogen peroxide levels in liver were measured by Wolff’s method [21]. FOX 1 (Ferrous Oxidation with Xylenol orange) reagent was prepared as the following mixture: 100 µM xylenol orange, 250 µM ammonium ferrous sulfate, 100 mM sorbitol, and 25 mM H_2_SO_4_. Fifty microliters of test sample was added to 950 µL FOX 1 reagent, vortexed, and incubated at room temperature for a minimum of 30 min at which time color development is virtually complete. The absorbance was read at 560 nm and the standard was linear in the 0∼5 µM concentration range. The hepatic thiobarbituric acid-reactive substances (TBARS) concentration, as a marker of lipid peroxide production, was measured spectrophotometrically by the method of Ohkawa et al. [22].

### Hepatic Morphology

The livers were removed from the mice and fixed in a buffer solution of 10% formalin. Fixed tissues were processed routinely for paraffin embedding, and 4-µm sections were prepared and stained with hematoxylin eosin (H&E); stained areas were viewed using an optical microscope with a magnifying power of ×200.

### Liver Enzyme Analyses

The hepatic cytosolic, mitochondrial and microsomal preparations were performed according to Hulcher and Oleson [23] with a slight modification, and the protein concentration was determined using Bradford’s method [24].

The glucokinase (GK) activity was determined using a spectrophotometric continuous assay as described by Davidson and Arion [25] and Newgard et al. [26] with a slight modification, in which the formation of glucose-6-phosphate was coupled to its oxidation by glucose-6-phosphate dehydrogenase and NAD^+^ at 37°C. The glucose-6-phosphatase (G6Pase) activity was determined using the method of Alegre et al. [27] with a slight modification. The reaction mixture contained 40 mmol/L sodium Hepes (pH 6.5), 14 mmol/L glucose-6-phosphate, 18 mmol/L EDTA, both previously adjusted to pH 6.5, 2 mmol/L NADP^+^, 0.6 IU/mL mutarotase, and 0.6 IU/mL glucose dehydrogenase. The phosphoenolpyruvate carboxykinase (PEPCK) activity was monitored in the direction of oxaloacetate synthesis using the spectrophotometric assay developed by Bentle and Lardy [28] with a slight modification. A 1 mL final volume of the purified enzyme was pipetted with a reaction mixture (pH 7.0) containing 77 mmol/L sodium Hepes, 1 mmol/L IDP, 1 mmol/L MnCl_2_, 1 mmol/L dithiothreitol, 0.25 mmol/L NADH, 2 mmol/L phosphoenolpyruvate, 50 mmol/L NaHCO_3_, and 7.2 units of malic dehydrogenase into a Eppendorf tube. The enzyme activity was then measured for 2 min at 25°C based on a decrease in the absorbance at 340 nm. The fatty acid synthase (FAS) activity was measured according to the method of Carl et al. [29] by monitoring the malonyl-CoA-dependent oxidation of NADPH at 340 nm, where the activity was represented by the oxidized NADPH nmol/min/mg protein. The phosphatidate phosphohydrolase (PAP) activity was determined using the method of Walton and Possmayer [30]. The carnitine palmitoyl transferase (CPT) activity was determined according to the method of Markwell et al. [31] and the results were expressed as nmol/min/mg protein. The fatty acid β-oxidation was determined using the method of Lazarow [32] by monitoring the reduction of NAD to NADH at 340 nm, where the activity was expressed as the reduced NAD nmol/min/mg protein. The 3-hydroxy-3-methylglutaryl-coenzyme reductase (HMGR) activity was measured in the microsomes with [^14^C]-HMG-CoA as the substrate based on a modification of the method of Shapiro et al. [33], where the activity was expressed as the synthesized mevalonate pmol/min/mg protein. The acyl CoA: cholesterol acyltransferase (ACAT) activity in the microsomes was determined by the rate of incorporation of [^14^C]-Oleoyl CoA into cholesterol ester fractions, as described by Erickson et al. [34], where the activity was expressed as the synthesized cholesteryl oleate pmol/min/mg protein. Superoxide dismutase (SOD) activity was spectrophotometrically measured by the inhibition of pyrogallol autoxidation at 420 nm for 10 min according to the method of Marklund and Marklund [35]. One unit was determined as the amount of enzyme that inhibited the oxidation of pyrogallol by 50%. Catalase (CAT) activity was measured using Aebi’s [36] method with a slight modification, in which the disappearance of hydrogen peroxide was monitored at 240 nm for 5 min using a spectrophotometer. A molar extinction coefficient of 0.041 mM^−1^cm^−1^ was used to determine CAT activity. Glutathione peroxidase (GPX) activity was measured using the spectrophotometric assay at 25°C, as described previously by Paglia and Valentine’s [37] method with a slight modification. The reaction mixture contained 2.525 mL of a 0.1 M of Tris-HCl (pH 7.2) buffer, 75 µL of 30 mM glutathione, 100 µL of 6 mM NADPH, and 100 µL of glutathione reductase (0.24 unit). One hundred microliters of the solution was added to 2.8 mL of the reaction mixture and incubated at 25°C for 5 min. The reaction was initiated by adding 100 µL of 30 mM H_2_O_2_ and the absorbance measured at 340 nm for 5 min. A molar extinction coefficient of 6.22 mM^−1^cm^−1^ was used to determine GPX activity.

### RNA Isolation and mRNA Expression Analysis

Total RNA was isolated from the livers by the guanidine thiocyanate-phenol method of Chomzynski and Sacchi [38]. For Northern blotting, the total RNA (20 µg) was separated on a 0.9% agarose gel containing 2.2 M formaldehyde and transferred to Nytran-Plus membranes (Schleicher & Schuell, Dassel, Germany). The membranes were then hybridized with a [^32^P]-labeled cDNA probe, washed at room temperature with 2× sodium chloride sodium citrate (SSC) containing 0.1% SDS followed by two washes at 65°C with 0.2× SSC containing 0.1% SDS, and exposed to X-ray film with an intensifying screen at −70°C. Thereafter, DNA probes were prepared from the mouse liver RNA using RT-PCR with the following primers: for GK, 5'-TTCACCTTCTCCTTCCCTGTAAGGC-3' and 5'-TACCAGCTTGAGCAGCACAAGTCG-3'; for G6Pase, 5'-AAGACTCCCAGGACTGGTTCATCC-3' and 5'-TAGCAGGTAGAATCCAAGCG CG-3'; for PEPCK, 5'-TGCTGATCCTGGGCATAACTAACC-3' and 5'-TGGGTACTCCTTCTGGAGATTCCC-3'; for Cu/Zn SOD, 5′-AGGATTAACTGAAGGCGAGCAT-3′ and 5′-TCTACAGTTAGCAGGCCAGCA G-3′; for CAT, 5′-ACGAGATGGCACACTTTGACAG-3′ and 5′-TGGGTTTCTCTTCTGGCTATGG-3′; for GPX, 5′-AAGGTGCTGCTCATTGAGAATG-3′ and 5′-CGTCTGGACCTACCAGGAACTT-3′; and for glyceraldehyde-3-phosphate dehydrogenase (GAPDH), 5'-TTGAAGGGTGGAGCCAAACG-3' and 5'-AGTGGGAGTTGCTGTTGAAGTCG-3'. The intensities of the mRNA bands were quantified using a Bio Image Whole Band Analyzer (50S, B.I. System Co., USA) and subsequently normalized based on the intensity of the respective GAPDH mRNA bands. For real-time PCR, complementary DNA was synthesized using Moloney murine leukemia virus reverse transcriptase (Fermentase, Burlington, ON, Canada), random hexamers, deoxyribonucleoside triphosphates, and 5 µg of total RNA. After first-strand complementary DNA synthesis, the RNA expression was quantified by a real-time quantitative PCR using SYBR green PCR reagnets (Applied Biosynstems, Foster City, CA) and the SDS7000 sequence-detection system (Applied Biosystems). Transcripts of the housekeeping gene GAPDH in the same incubations were used for normalization. The primer sequences were as follows: for ATP-citrate lyase (ACL), 5′–CAGCAAGCACTGTCAGAATA–3′ and 5′–TTAAAACTTGCATTCCCTTC–3′; for CPT, 5′-ATCTTCTAATCCCACCCAGT-3′ and 5′-AAAGCACCCATTACTTGAGA-3′; for diacylglycerol transferase (DGAT), 5′-ACTCCATCATGTTCCTCAAG-3′ and 5′-CTCTGCTTGTGAGAAGGAAC-3′; for PAP, 5′-GGGTTCTACTGTGGAGATGA-3′ and 5′-TGACAGTAGCTGTGATGATGA-3′; for peroxisome proliferator activated receptorγ (PPARγ), 5′–AGAGTCTGCTGATCTGCGAGC–3′ and 5′–TTCCTGTCAAGATCGCCCTC–3′; for stearoyl-CoA desaturase-1 (SCD-1), 5′–TAAGCTCAGTCTCACTCCTT–3′ and 5′–AAAAGATTTCTGCAAACCAA–3′.

### Statistical Analysis

All data were presented as the mean ± S.E. Statistical analyses were performed using the statistical package for the social science software (SPSS) program. Student’s t-test was used to assess the differences between the groups. Statistical significance was considered at p<0.05.

## Results

### PL Ameliorates Hyperglycemia and Insulin Resistance and Increased Plasma Adiponectin Level

**Figure 1:**
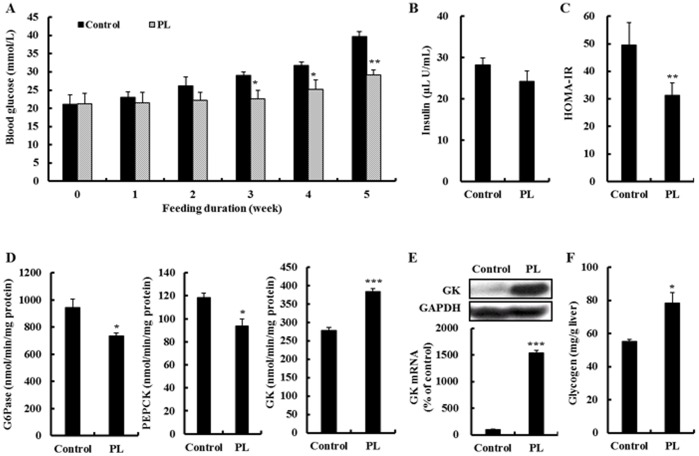
Effect of supplementation with powdered persimmon leaf on the glucose regulation in C57BL/KsJ-*db/db* mice. A; fasting blood glucose level. B&C plasma insulin level and HOMA-IR. D&E activity and/or mRNA expression of enzymes for hepatic gluconeogenesis and glucose utilization. The mRNA levels were analyzed by Northern blot analysis using GK probes and normalized to an internal control (GAPDH). Three independent analyses were performed. F; hepatic glycogen content. Values are the mean ± S.E., n = 10. *p<0.05, **p<0.01, ***p<0.001. GAPDH; glyceraldehyde-3-phosphate dehydrogenase, G6Pase; glucose-6-phosphatase, GK; glucokinase, HOMA-IR; homeostatic index of insulin resistance, PEPCK; phosphoenolpyruvate carboxykinase, PL; powdered persimmon leaf.

First, we examined the effect of PL on hyperglycemia and insulin resistance in type 2 diabetic *db/db* mice. All *db/db* mice were diabetic when the experiment began, as indicated by the fasting blood glucose level (≥21.06 mmol/L) (Fig. 1A). The supplementation of PL suppressed the increase of the fasting blood glucose level from the 3^rd^ week to the 5^th^ week in the *db/db* mice. The plasma insulin level was not affected by PL supplementation, while PL improved HOMA-IR in the *db/db* mice (Fig. 1B&C). Furthermore, PL did not alter the plasma leptin level, yet it significantly increased the plasma adiponectin level (Table 1). There was no significant difference in food intake, body weight and body fat mass between the groups (data not shown).

**Table 1:** Effects of supplementation with persimmon leaf on the levels of lipids and adipokines and on the activity of paraoxonase in plasma of C57BL/KsJ-*db/db* mice.

	Control	PL
Free fatty acid (mmol/L)	1.54±0.03	1.56±0.07
Triglyceride (mmol/L)	3.32±0.19	2.09±0.25*
Total cholesterol (mmol/L)	5.62±0.12	4.73±0.2^**^
HDL-cholesterol (mmol/L)	1.06±0.07	1.44±0.06*
HTR (%)	18.86±0.93	30.35±1.17^***^
Atherogenic index	4.31±0.02	2.29±0.13^***^
Adiponectin (µg/mL)	2.26±0.22	3.72±0.14^**^
Leptin (ng/mL)	49.1±3.16	50.5±4.07
PON (µmol/min/mL)	1.30±0.02	1.62±0.05*

Data are Mean ± S.E. (n = 10).

* p<0.05, ^**^ p<0.01, ^***^ p<0.001 vs. control group as determined by student’s t-test.

HTR (%); [(HDL-cholesterol)/(Total cholesterol)]×100, Atherogenic index; [(Total cholesterol)– (HDL-cholesterol)]/(HDL-cholesterol), PON; paraoxonase.

### PL Increases Glycogen Content and GK Activity and its Gene Expression and Decreases G6Pase and PEPCK Activity in the Liver

To examine how PL ameliorated hyperglycemia, we determined the activity of the hepatic glucose-regulating enzymes, their gene expression and glycogen content (Fig. 1D–F). The GK activity and mRNA expression as well as glycogen content were significantly higher in the liver of the PL-supplemented *db/db* mice compared to the control *db/db* mice. In contrast, the supplementation of PL significantly lowered the activity of hepatic gluconeogenic enzymes, G6Pase and PEPCK. However, no difference was observed in the mRNA expression of hepatic G6Pase and PEPCK between the groups (data not shown).

### PL Attenuates Dyslipidemia and Hepatic Steatosis

**Figure 2:**
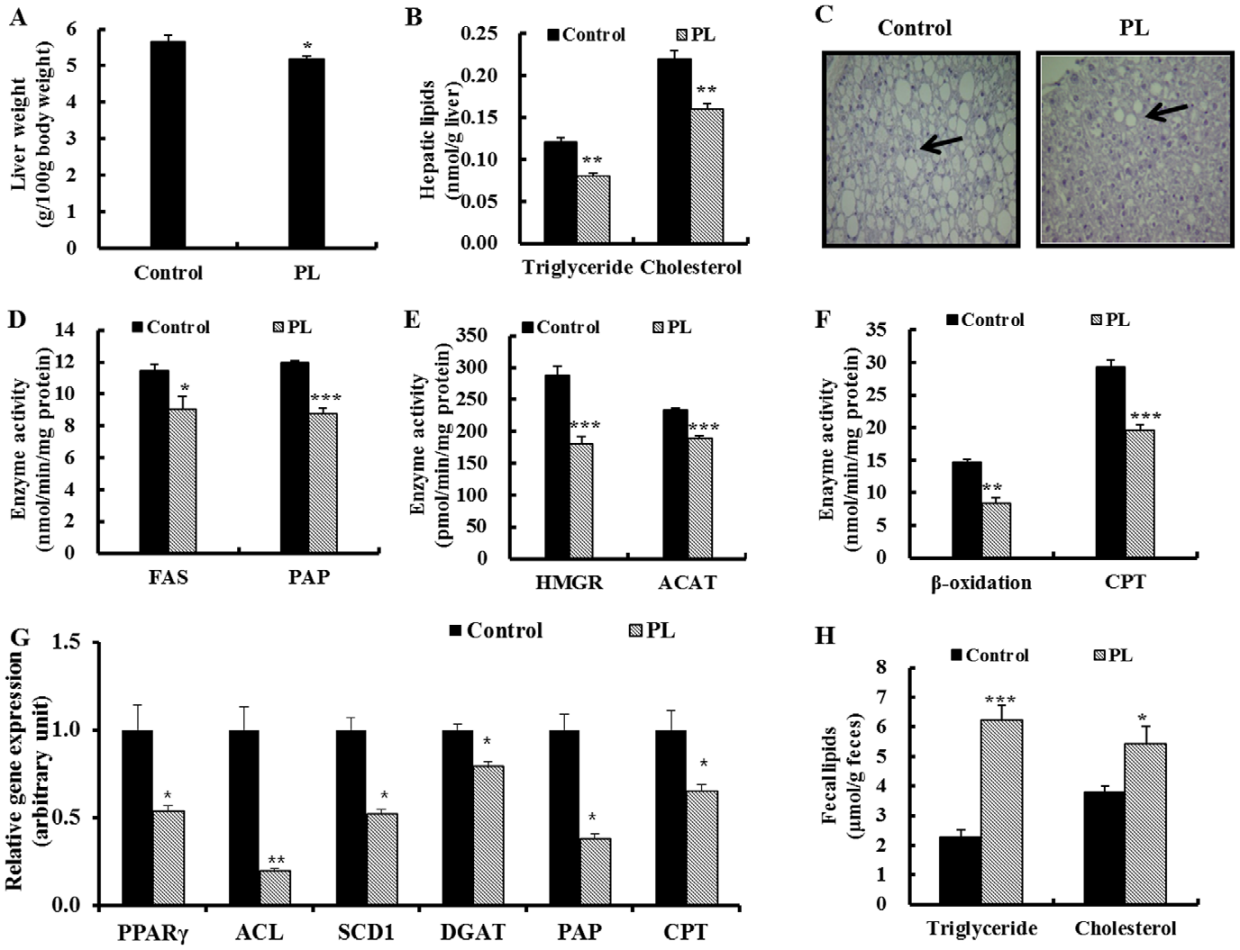
Effect of supplementation with powdered persimmon leaf on the lipid regulation in C57BL/KsJ-*db/db* mice. A; liver weight. B; hepatic triglyceride and cholesterol contents. C; hepatic morphology. Arrows indicate hepatic lipid droplets. H&E staining. Original magnification ×200. D; activity of the enzymes for hepatic fatty acid synthesis and esterification. E; activity of the enzymes for hepatic cholesterol synthesis and esterification. F; activity of the enzymes for hepatic fatty acid oxidation and uptake. G; mRNA expression of lipogenic transcription factor and its target gene. The mRNA levels were analyzed by real-time PCR and normalized to an internal control (GAPDH). H; fecal lipids content. Values are the mean ± S.E., n = 10. *p<0.05, **p<0.01, ***p<0.001. ACAT; acyl CoA: cholesterol acyltransferase, ACL; ATP-citrate lyase, CPT; carnitine palmitoyl transferase, DGAT; glyceraldehyde-3-phosphate dehydrogenase, FAS; fatty acid synthase, HMGR; 3-hydroxy-3-methylglutaryl-coenzyme, PAP; phosphatidate phosphohydrolase, PL; powdered persimmon leaf, PPARγ; peroxisome proliferator activated receptor γ, SCD1; steraroyl-CoA desaturase-1.

Next, we evaluated the effect of PL on dyslipidemia and hepatic steatosis in the *db/db* mice. The plasma triglyceride and total cholesterol concentrations were significantly lower in the PL group than in the control group, whereas the plasma HDL-cholesterol concentration was significantly higher in the PL group (Table 1). Thus, an increased HTR and decreased atherogenic index were observed in the PL group. Furthermore, PL supplementation significantly lowered hepatic triglyceride and cholesterol contents compared to the control *db/db* mice, and liver weight was significantly lower in the PL group (Fig. 2A&B). Histological analysis of the liver also revealed lower numbers and size of hepatic lipid droplets in the PL-supplemented *db/db* mice compared to the control *db/db* mice (Fig. 2C).

### PL Inhibits Hepatic Lipogenic Gene Expression and Enzymes Activity as well as Transcriptional Factor PPARγ mRNA Expression and Increases Fecal Lipids

To clarify the mechanism in which PL decreased the plasma and hepatic lipids levels, we examined the hepatic lipid-regulating enzyme activity and gene expression along with fecal lipids levels. The activity of the hepatic lipogenic enzymes, FAS, PAP, HMGR and ACAT, were significantly lower in the PL-supplemented *db/db* mice compared to the control *db/db* mice (Fig. 2D&E). PL also down-regulated mRNA expression of lipogenic transcription factor (PPARγ) and its target genes (ACL, SCD1, DGAT, PAP) in the liver (Fig. 2G). Moreover, the supplementation of PL significantly increased the fecal triglyceride and cholesterol content (Fig. 2H). The fatty acid oxidation as well as CPT activity and mRNA level were markedly lower in the PL group compared to the control group (Fig. 2F&G).

### PL Improves Plasma and Hepatic Oxidative Stress

**Figure 3:**
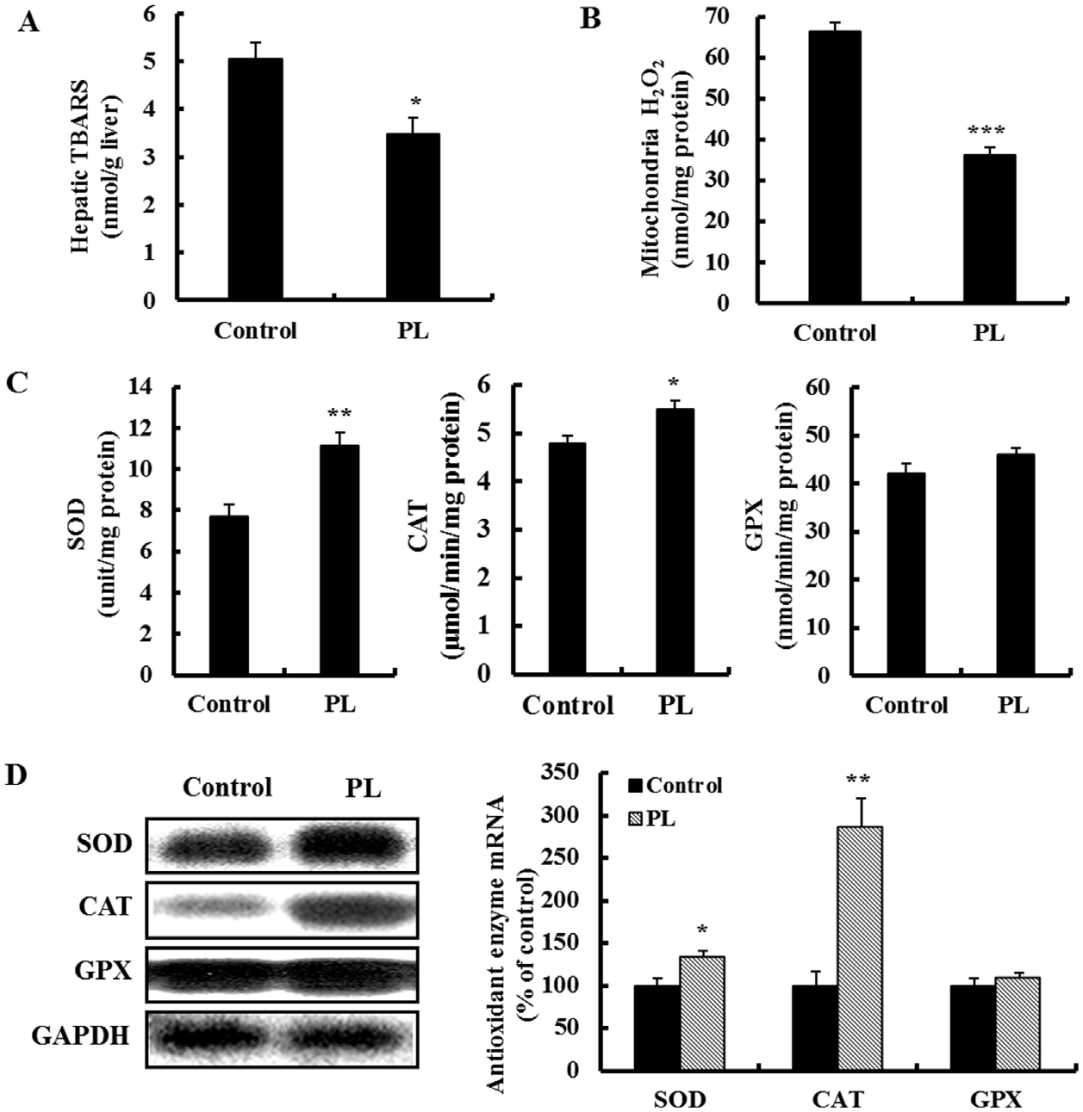
Effect of supplementation with powdered persimmon leaf on hepatic oxidative stress in C57BL/KsJ-*db/db* mice. A; hepatic TBARS level. B; hepatic mitochondria hydrogen peroxide content. C; hepatic antioxidant enzyme activity. D; hepatic antioxidant gene expression. The mRNA levels were analyzed by Northern blot analysis using SOD, CAT and GPX probes and normalized to an internal control (GAPDH). Three independent analyses were performed. Values are the mean ± S.E., n = 10. *p<0.05, **p<0.01, ***p<0.001. CAT; catalase, GPX; glutathione peroxidase, GAPDH; glyceraldehyde-3-phosphate dehydrogenase, PL; powdered persimmon leaf, SOD; superoxide dismutase.

We next examined the effect of PL on hepatic lipid peroxidation, hydrogen peroxide levels, and antioxidant enzymes activities and their mRNA expression, which influence the regulation of glucose and lipid metabolism in the *db/db* mice. The lipid peroxidation and mitochondrial hydrogen peroxide levels were significantly lower in the liver of the PL-supplemented *db/db* mice compared to the control *db/db* mice (Fig. 3A&B). Hepatic GPX activity and mRNA expression was not different between the groups; however, SOD and CAT activities and their mRNA expression were significantly increased by PL supplementation (Fig. 3C&D). Furthermore, PL significantly increased the activity of PON in plasma (Table 1).

**Figure 4:**
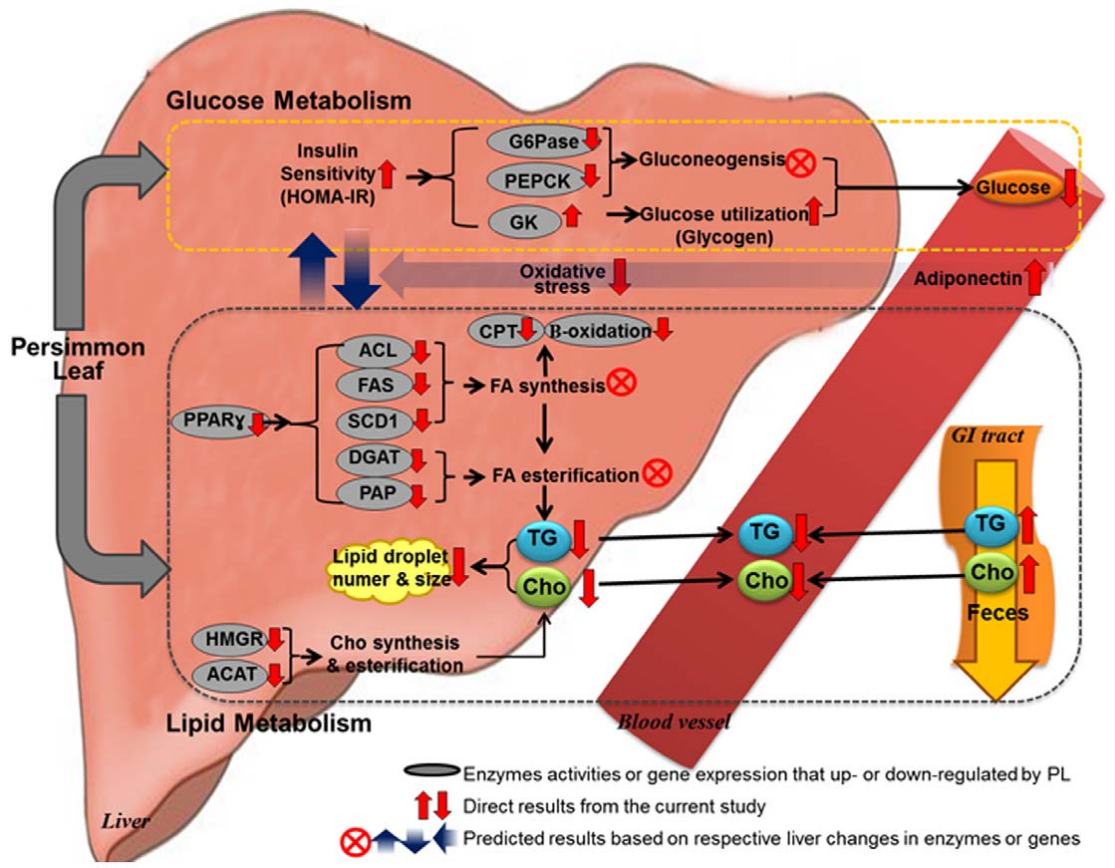
Proposed mechanism of PL on the glucose and lipid lowering action in C57BL/KsJ-*db/db* mice. PL improved HOMA-IR, which may activate glucokinase activity and its mRNA expression and inhibit gluconeogenic enzymes activity in the liver, resulting in lowered blood glucose level. The enhanced hepatic insulin sensitivity may be related to the improved hepatic steatosis and dyslipidemia, since PL led to lower plasma and hepatic lipid levels via reduction of transcription factor PPARγ, lipogenic gene expression and enzyme activity with a simultaneous increase in fecal lipids excretion. Furthermore, PL ameliorated oxidative stress and increased adiponectin secretion, which may be also associated with improved insulin sensitivity, hepatic steatosis and dyslipidemia. ACAT; acyl CoA: cholesterol acyltransferase, ACL; ATP-citrate lyase, Cho; cholesterol, CPT; carnitine palmitoyl transferase, DGAT; glyceraldehyde-3-phosphate dehydrogenase, FA; fatty acid, FAS; fatty acid synthase, G6Pase; glucose-6-phosphatase, GK; glucokinase, GI tract; gastrointestinal tract, HMGR; 3-hydroxy-3-methylglutaryl-coenzyme, HOMA-IR; homeostatic index of insulin resistance, PAP; phosphatidate phosphohydrolase, PL; powdered persimmon leaf, PEPCK; phosphoenolpyruvate carboxykinase, PPARγ; peroxisome proliferator activated receptor γ, SCD1; steraroyl-CoA desaturase-1, TG; triglyceride.

## Discussion

Type 2 diabetes is a metabolic syndrome with diverse pathological manifestations and is often associated with abnormal glucose and lipid metabolism. Previously, we reported that the supplementation of PL that is rich in fiber and phenolic compounds suppressed the body weight gain and lowered plasma and hepatic lipid levels in rats fed a high-fat diet [10]. Furthermore, several *in vitro* studies showed the possible beneficial effect of PL on glucose regulation. For example, PL inhibited enzymes involved in the digestion of carbohydrates (α-amylase and α-glucosidase) and the regulation of insulin signaling (protein tyrosine phosphatase 1B) [5]–[7]. However, no report has been published on the *in vivo* anti-diabetic effects of PL in type 2 diabetes. In this study, we firstly demonstrated that dietary supplementation of PL for 5 weeks ameliorated hyperglycemia, dyslipidemia and hepatic steatosis in type 2 diabetic mice, at least in part, by regulation of hepatic glucose and lipid metabolism and antioxidant status.

*db/db* mice are a widely used genetic model of type 2 diabetes since they exhibit most of the human characteristics of type 2 diabetes, including hyperglycemia, dyslipidemia and insulin resistance [11]. A recent study suggests that defects in hepatic insulin signaling contribute to the development of diabetes in C57BL/KsJ-*db/db* mice [39]. The liver is a major insulin-sensitive organ responsible for maintaining glucose and lipid homeostasis. A failure of insulin to increase hepatic glucose utilization and to suppress hepatic endogenous glucose production is a major factor contributing to hyperglycemia in diabetes [40]. The key enzyme responsible for the regulation of glucose utilization is GK that catalyzes glucose phosphorylation as the first step of storage of glucose as glycogen and glucose disposal by glycolysis [41]. Conversely, PEPCK and G6Pase are rate-controlling enzymes of gluconeogenesis in the liver [42]. We found that in the *db/db* mice, the supplementation of PL significantly decreased the fasting blood glucose level and HOMA-IR index, which primarily reflects hepatic insulin resistance [43]. These changes were accompanied by decreases in hepatic G6Pase and PEPCK activity and increases in hepatic GK activity along with glycogen content. Thus, these observations suggest that PL can promote hepatic insulin sensitivity and thus effectively regulate the activity of enzymes involved in hepatic glucose homeostasis, leading to lower blood glucose level in *db/db* mice.

The regulation of GK activity is primarily due to changes in the transcription of its gene [44]. We also found that the change in GK activity by PL was accompanied by its increased transcriptional level. In contrast, the gene expression of hepatic G6Pase and PEPCK was not affected. Similarly, a lack of regulation of G6Pase and PEPCK gene expression was reported following treatment with some phenolic compounds in rat hepatocytes, despite a significant reduction in glucose production and enhanced hepatic GK mRNA expression [45]. In addition, it is known that the suppression of hepatic glucose production by metformin results from the inhibition of G6Pase activity along with an increase in glycogen stores with minimal effects on the gluconeogenic gene expression in the livers of rats [46], [47]. Thus, we think that PL may act mainly by suppressing the activity of hepatic gluconeogenic enzymes independent of the transcriptional repression of gluconeogenic genes. Since elevated GK expression led to a reduced endogenous glucose production in the liver [48], it is possible that the observed decrease in activity of gluconeogenic enzymes in PL-supplemented *db/db* mice is related to the inhibition of the substrate flux through GK activation.

On the other hand, some studies have raised concerns about the manipulation of GK activator for diabetes treatment since a decline in glucose level in response to hepatic GK overexpression is accompanied by an increase in circulating lipids and hepatic lipogenesis [49], [50]. However, several lines of evidence suggest that hepatic GK activation does not alter plasma and hepatic lipid metabolism in normal and high-fat fed animals [49], [51]. The present results also showed that PL induced a marked decrease in triglyceride and cholesterol accumulation in the liver, together with the inhibition of activity of hepatic lipogenic enzymes involved in the synthesis and esterification of fatty acid (FAS, PAP) or cholesterol (HMGR, ACAT), which may subsequently reduce the formation of lipid droplets within hepatocytes and the secretion of triglycerides and cholesterol into the blood. Simultaneously, this effect could be related to the down-regulated expression of several lipogenic genes (ACL, SCD1, PAP, DGAT) as well as key transcription factor (PPARγ) in the liver. Normally, PPARγ is expressed at very low levels in the liver, but its expression is dramatically increased in animal model with insulin resistance and hepatic steatosis such as *db/db* mice [52]. The genetic deletion of hepatic PPARγ protected against hepatic steatosis in high fat diet-induced obese mice [53]. We also found that PL appeared to facilitate fecal excretion of triglycerides as well as cholesterol in the *db/db* mice, in accordance with our previous data on high-fat fed rats [10]. Accordingly, PL seemed to lower plasma and hepatic lipid accumulation by decreasing hepatic lipogenesis and increasing fecal lipids. Since inhibition of FAS induces an increase in hepatic malonyl-CoA which is a potent inhibitor of CPT [54], [55], a key enzyme involved in mitochondrial fatty acids uptake for oxidation [56], the reduced activity of hepatic fatty acid oxidation and CPT could be a secondary consequence of the decrease in hepatic FAS activity. Also, the reduced fatty acid oxidation might be associated with activated glucose utilization and a reduction in glucose production in the liver [57].

In addition to its role in regulating lipogenesis, it is known that PPARγ is required for transcription of the PEPCK gene in adipocytes [58]. PPARγ agonists such as pioglitazone and rosiglitazone were potent inducer of PEPCK gene transcription and enzymatic activity in adipose tissue of obese Zucker rats [59], [60]. Moreover, hepatocyte specific PPARγ-knockout mice showed reduced serum glucose level and PEPCK mRNA expression [53]. However, liver-specific disruption of PPARγ in leptin-deficient mice dramatically increased basal endogenous glucose production [61]. Also, PPARγ agonist, troglitazone, inhibited the expression of PEPCK gene by a PPARγ-independent, antioxidant-related mechanism, and other PPARγ agonists, including rosiglitazone and ciglitazone, had little effect on PEPCK gene expression in hepatocyte [62], suggesting that the regulation of PEPCK by PPARγ is cell-specific [63]. We also observed that PEPCK mRNA expression was not altered by PL supplementation, although the PL down-regulated hepatic PPARγ and its target lipogenic genes expression. In fact, the regulation of PEPCK gene transcription coordinated by the action of a number of transcriptional factors and various hormones, including insulin, glucocorticoids, retinoic acid, thyroid hormone, and cyclic AMP [64]–[66]. Thus, it is possible that the PEPCK gene expression in PL-supplemented *db/db* mice was controlled by cooperative interaction of multiple transcription factors and hormones involved in PEPCK gene regulation.

Oxidative stress has been implicated in the pathogenesis of diabetes and other metabolic syndrome, including fatty liver disease and cardiovascular disease. In particular, the mitochondria is a major source of reactive oxygen species (ROS), and the ROS generated from the mitochondria damages proteins, DNA, and lipids in the membrane components, which results in mitochondrial dysfunction [67], [68]. Under normal physiological conditions, ROS are continuously produced, and oxidative damage induced by ROS can be prevented by antioxidant enzymes, where superoxide anion is rapidly converted by SOD into hydrogen peroxide, which is eliminated by CAT and GPX. However, an imbalance between ROS production and antioxidant capacity can induce cell damage associated with diabetes. Increased ROS levels were observed in the liver of *db/db* mice [69] and the expression of the antioxidant gene was down-regulated in the liver of type 2 diabetic rats [70]. In contrast, overexpression of SOD or CAT protected against hepatic oxidative injury in the livers of *db/db* mice [68] or HepG2 cells [71]. We found that PL up-regulated the mRNA expression of SOD and CAT in the liver. In parallel with the enhanced antioxidant gene expression, hepatic SOD and CAT activity was increased in the PL-supplemented *db/db* mice, suggesting that the elevated SOD and CAT expression may be regulated at the transcriptional level. Thus, the decreased level of mitochondria hydrogen peroxide in the liver of PL-supplemented mice may be attributed to the improved hepatic antioxidant capacity, which may contribute to the decreased hepatic lipid peroxidation and provide protection against hepatic oxidative stress in type 2 diabetes.

We also found that PL significantly increased the plasma PON activity as well as the plasma HDL-cholesterol and adiponectin levels. Plasma PON is another antioxidant enzyme contained in plasma HDL, which protects LDL and HDL from oxidation by ROS, and possesses other multiple anti-atherogenic activities [72]. Serum PON activity is low in patients with diabetes and it has potential as a marker for atherosclerosis in diabetes [73]. Along with this enzyme activity, HDL-cholesterol is an independent predictor of atherosclerotic cardiovascular complications. Furthermore, the adiponectin concentration is positively correlated with HDL cholesterol and negatively associated with HOMA-IR, independent of age and BMI, in type 2 diabetic subjects [74]. Taken together, our findings suggest the potential protective effects of PL on atherosclerotic cardiovascular complications in type 2 diabetes.

In conclusion, our results show that dietary PL improved hyperglycemia by alterations in activity and/or mRNA expression of hepatic enzymes involved in glucose utilization and glucose production (Fig. 4). Furthermore, PL ameliorated dyslipidemia and hepatic steatosis through a combined decrease in hepatic lipogenesis and an increase in the excretion of fecal lipids, which seemed to be related to the enhanced responsiveness of the liver to insulin (Fig. 4). The beneficial metabolic effects were also related to decreased plasma and hepatic oxidative stress as well as increased adiponectin secretion (Fig. 4). Thus, we believe that PL is a promising anti-diabetic compound that will be helpful for improving type 2 diabetes, although further study is required to identify its active components that mediate the hypoglycemic, hypolipidemic and hepatoprotective effects of PL.
